# Persistent memory despite rapid contraction of circulating T Cell responses to SARS-CoV-2 mRNA vaccination

**DOI:** 10.3389/fimmu.2023.1100594

**Published:** 2023-02-13

**Authors:** Ellie Taus, Christian Hofmann, F. Javier Ibarrondo, Laura S. Gong, Mary Ann Hausner, Jennifer A. Fulcher, Paul Krogstad, Scott G. Kitchen, Kathie G. Ferbas, Nicole H. Tobin, Anne W. Rimoin, Grace M. Aldrovandi, Otto O. Yang

**Affiliations:** ^1^ Department of Molecular and Medical Pharmacology, David Geffen School of Medicine, University of California, Los Angeles, Los Angeles, CA, United States; ^2^ Department of Medicine, David Geffen School of Medicine, University of California Los Angeles, Los Angeles, CA, United States; ^3^ Department of Microbiology, Immunology, and Molecular Genetics, David Geffen School of Medicine, University of California Los Angeles, Los Angeles, CA, United States; ^4^ Department of Pediatrics, David Geffen School of Medicine, University of California Los Angeles, Los Angeles, CA, United States; ^5^ Fielding School of Public Health, University of California Los Angeles, Los Angeles, CA, United States

**Keywords:** SARS-CoV-2, cellular immunity, T cells, elispot, intracellular cytokine staining, SARS-CoV-2 mRNA vaccines, T cell memory

## Abstract

**Introduction:**

While antibodies raised by SARS-CoV-2 mRNA vaccines have had compromised efficacy to prevent breakthrough infections due to both limited durability and spike sequence variation, the vaccines have remained highly protective against severe illness. This protection is mediated through cellular immunity, particularly CD8+ T cells, and lasts at least a few months. Although several studies have documented rapidly waning levels of vaccine-elicited antibodies, the kinetics of T cell responses have not been well defined.

**Methods:**

Interferon (IFN)-γ enzyme-linked immunosorbent spot (ELISpot) assay and intracellular cytokine staining (ICS) were utilized to assess cellular immune responses (in isolated CD8+ T cells or whole peripheral blood mononuclear cells, PBMCs) to pooled peptides spanning spike. ELISA was performed to quantitate serum antibodies against the spike receptor binding domain (RBD).

**Results:**

In two persons receiving primary vaccination, tightly serially evaluated frequencies of anti-spike CD8+ T cells using ELISpot assays revealed strikingly short-lived responses, peaking after about 10 days and becoming undetectable by about 20 days after each dose. This pattern was also observed in cross-sectional analyses of persons after the first and second doses during primary vaccination with mRNA vaccines. In contrast, cross-sectional analysis of COVID-19-recovered persons using the same assay showed persisting responses in most persons through 45 days after symptom onset. Cross-sectional analysis using IFN-γ ICS of PBMCs from persons 13 to 235 days after mRNA vaccination also demonstrated undetectable CD8+ T cells against spike soon after vaccination, and extended the observation to include CD4+ T cells. However, ICS analyses of the same PBMCs after culturing with the mRNA-1273 vaccine in vitro showed CD4+ and CD8+ T cell responses that were readily detectable in most persons out to 235 days after vaccination.

**Discussion:**

Overall, we find that detection of spike-targeted responses from mRNA vaccines using typical IFN-γ assays is remarkably transient, which may be a function of the mRNA vaccine platform and an intrinsic property of the spike protein as an immune target. However, robust memory, as demonstrated by capacity for rapid expansion of T cells responding to spike, is maintained at least several months after vaccination. This is consistent with the clinical observation of vaccine protection from severe illness lasting months. The level of such memory responsiveness required for clinical protection remains to be defined.

## Introduction

The mRNA vaccines against SARS-CoV-2 have had a remarkable impact reducing morbidity and mortality of the COVID-19 pandemic. They encode the spike protein to elicit two major classes of adaptive immune responses, including neutralizing antibodies and T cells. These responses appear to have rather distinct roles in protection, with antibodies predominantly reducing early symptomatic infection and T cells (particularly the CD8^+^ cytotoxic subset) preventing severe illness and death after infection (1–4).

It has become clear that vaccine protection has limited durability, resulting in recommendations for intermittent “booster” vaccinations (5). Many studies have demonstrated the rapid decay of anti-spike antibodies elicited by vaccination (6–15), as well as those from SARS-CoV-2 infection (16–26). This is likely a factor in the high frequency of “breakthrough” infections and re-infections among vaccinees (27–32) and COVID-19-recovered persons (33–37), although variation of the spike sequence (particularly the receptor binding domain that is the main target of neutralizing antibodies) is a major contributor (13, 29, 38–42). Vaccine protection from severe illness has been more durable (43–45), which might be due at least in part to cellular immunity and epitope sequences being less affected by spike sequence variation than neutralizing antibodies (38, 46–50). However, protection by vaccines against severe illness also appears to decline with time (31, 43, 51–53), suggesting the waning of cellular immunity as well.

The contribution of waning cellular immunity is unclear, and the kinetics of T cell responses are not well understood. Early trials of mRNA-1273 (54) and BNT162b2 (55) mRNA vaccines documented cellular immune responses, subsequently confirmed by several groups that have described both CD4^+^ and CD8^+^ T cell anti-spike responses elicited by vaccination (56–58). Detailed data on the long-term persistence of these responses and those from SARS-CoV-2 infection have been limited, although some reports have suggested at least some waning of both vaccine-elicited (14, 59, 60) and infection-elicited (61, 62) responses over months. Here we investigate the durability of cellular immune responses against SARS-CoV-2 spike protein, comparing those elicited by mRNA vaccines versus SARS-CoV-2 infection.

## Methods

### Study participants

All participants gave written informed consent through an institutional review board-approved protocol at the University of California Los Angeles. Persons with immunocompromising conditions such as diabetes mellitus, HIV-1 infection, or iatrogenic immunosuppression were excluded. Vaccinee participants had no prior history of COVID-19, and negative antibodies against the receptor binding domain (RBD) of the SARS-CoV-2 spike protein before vaccination. Participants who were COVID-19-recovered persons had been infected in January 2021 or earlier.

### Samples

PBMC were separated by Ficoll density gradient centrifugation and cryopreserved viably in heat-inactivated fetal calf serum with 10% dimethylsulfoxide for storage in vapor phase liquid nitrogen. They were thawed immediately before experimental use.

### CD8^+^ T cell IFN-γ ELISpot assays

Spike-specific CD8^+^ T cell responses were quantified using expanded CD8^+^ T cells as previously described in detail (61) and shown to produce results closely reflecting measurements using unexpanded peripheral blood CD8^+^ T cells (63–65). In brief, peripheral blood mononuclear cells (PBMC) were non-specifically expanded for approximately 14 days using a CD3:CD4 bi-specific antibody (generous gift of Dr. Johnson Wong). These were screened in a standard ELISpot assay against 12 peptide pools of 15-mer synthetic peptides spanning the SARS-CoV-2 spike protein (BEI Resources catalog #NR-52402). Negative control wells included triplicate wells with no peptide, duplicate wells with pooled peptides spanning the SARS-CoV-2 nucleocapsid protein, and duplicate positive control wells included phytohemagglutinin (PHA). Counts from each well were background subtracted using the average count from the negative control wells, and the total spike response was determined as the sum of all 12 peptide pool wells. Results totaling ≤ 50 spot forming cells (SFC) per million CD8^+^ T cells were considered negative, based on a prior ELISpot validation study (66).

### Anti-RBD antibody measurements

Serum immunoglobulin G SARS-CoV-2 spike RBD-specific antibodies were quantified as described in detail (6). Briefly, duplicate serum samples were added to 96-well microtiter plates that had been coated with recombinant RBD protein. After washing, goat anti-human IgG conjugated with horseradish peroxidase was added, followed by washing and addition of tetramethylbenzidine substrate. Measurements were performed at 450 and 650 nm, and the results were compared to a standard curve generated by a control titration of the anti-RBD monoclonal antibody CR3022 (Creative Biolabs, Shirley, NY). Serum anti-RBD IgG binding activity was expressed as equivalence to a concentration of CR3022.

### Assessment of spike-specific T cells by intracellular cytokine staining (ICS) flow cytometry

ICS staining and flow cytometry were performed as described in detail (61), except differing in the peptide target. In brief, PBMC were incubated with pooled overlapping 15-mer peptides spanning spike (67) containing 1µg/ml each peptide, with brefeldin A (catalog #00-4506-51, eBioscience, San Diego, CA) and monensin (#00-4505-51, eBioscience, San Diego, CA), followed by surface staining with CD3-Super Bright 436, CD8-Super Bright 600, CD4 PE-Cy7, and Fixable Aqua viability dye (catalog #62-0037-42/eBioscience/San Diego/CA, #63-0088-42/eBioscience/San Diego/CA, #25-0049-42/San Diego/CA, and #L34957/Invitrogen/Waltham/MA respectively), permeabilization (catalog #00-5523-00, eBioscience, San Diego, CA), and intracellular cytokine staining for IFN-γ-FITC, IL-2-PerCP-Cy5.5, IL-4-PE, and IL-10-APC (catalog #506504/Biolegend/San Diego/CA, #500322/Biolegend/San Diego/CA, # 130-091-647/Miltenyi Biotec/Bergisch Gladbach/Germany, and #506807/Biolegend/San Diego/CA respectively) followed by flow cytometric analysis.

### Culture of PBMC with mRNA-1273 vaccine for enriched detection of memory T cells targeting spike

When PBMC were utilized to measure anti-spike T cell responses by ICS immediately upon thawing, a portion was cultured with the mRNA-1273 vaccine *in vitro*. One to two million PBMC per well were cultured in RPMI 1640 (supplemented with L-glutamine, HEPES buffer, and antibiotic) with recombinant human IL-2 at 50U/ml (NIH AIDS Reagent Repository Program) and initially added mRNA-1273 vaccine (Moderna) at the specified concentration, in 24-well flat bottom tissue culture plates. Medium was replenished twice a week for about 14 days of culture, after which the cells were evaluated by ICS for anti-spike T cell responses as described above, with viable cryopreservation of a portion. If this analysis yielded fewer than 10,000 events in the CD4^+^ or CD8^+^ T cell compartments, ICS was repeated on the cryopreserved cells and weighted averaging was performed to combine the results.

## Results

### Longitudinal evaluation of CD8+ T cell responses by IFN-γ ELISpot assay after mRNA vaccination against SARS-CoV-2 demonstrates remarkably short-lived detection compared to natural infection, while antibody responses showed classical kinetics

**Figure 1:**
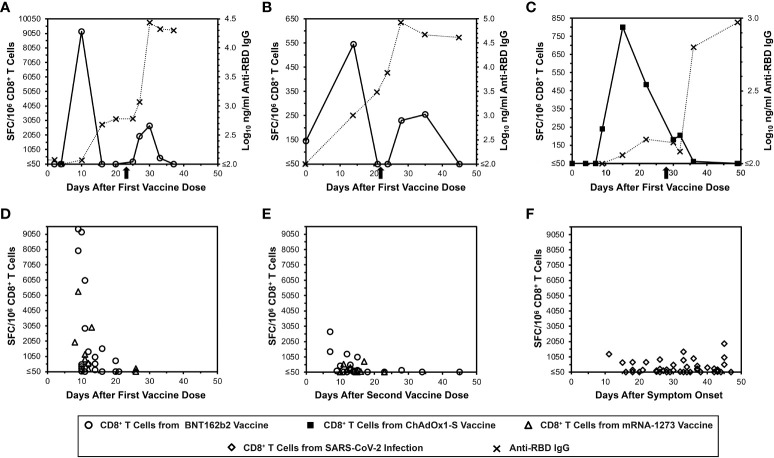
Transience of peripheral blood SARS-CoV-2 spike-specific CD8+ T cells elicited by mRNA vaccination compared to natural infection, as assessed by IFN-γ ELISpot. Spike-specific CD8+ T cells were assayed by IFN-γ ELISpot assay using pooled overlapping peptides.  **(A, B)** Serial CD8+ T cell responsesc against spike (open circles) and IgG responses against the spike RBD (Xs) are plotted for two SARS-CoV-2-naïve persons who received the BNT162b2 vaccine. The X-axis starts with the first vaccine dose, and the timing of the second dose is indicated by an arrow. **(C)** Serial CD8+ T cell responsesc against spike (closed squares) and IgG responses against the spike RBD (Xs) are plotted for a SARS-CoV-2-naïve person who received the ChAdOx1-S vaccine. The X-axis starts with the first vaccine dose, and the timing of the second dose is indicated by an arrow. **(D)** CD8+ T cell spike-specific responses are plotted for 25 persons who were SARS-CoV-2-naïve after the first vaccine dose with BNT162b2 (16 persons, 20 data points, circles) or mRNA-1273 (9 persons, 9 data points, triangles).  **(E)** CD8+ T cell spike-specific responses are plotted for 24 persons who were SARS-CoV-2-naïve after the second vaccine dose with BNT162b2 (15 persons, 20 data points, circles) or mRNA-1273 (9 persons, 9 data points, triangles).  **(F)** CD8+ T cell spike-specific responses are plotted for 45 COVID-19-recovered persons according to time after symptom onset (diamonds).

To demonstrate the acute kinetics of anti-spike CD8+ T cell responses to mRNA SARS-CoV-2 vaccination in detail, IFN-γ ELISpot assays were performed serially for SARS-CoV-2-naïve persons every two to four days after receiving BNT162b2 vaccination (**Figures 1A, B**). Detection of anti-spike responses was surprisingly short-lived, demonstrating sharp peaks lasting less than 10 days after each dose. However, humoral responses exhibited more typical kinetics; anti-RBD antibodies rose with persistence and progressive boosting after each dose. By comparison, a third person who got ChAdOx1-S vaccination (**Figure 1C**) showed different CD8+ T cell response kinetics, with a later initial anti-spike response that persisted to the second vaccine dose, although the second peak was minimal. In this person, the anti-RBD antibody level kinetics also evolved with similar kinetics to the mRNA vaccinees. These results suggested that mRNA vaccines yielded distinct kinetics compared to other vaccine platforms that yield CD8+ T cell responses.

### Cross-sectional evaluation of additional mRNA vaccinees confirms similar kinetics of CD8+ T cell responses, which differ from the kinetics after natural SARS-CoV-2 infection

More SARS-CoV-2-naïve mRNA vaccinees were evaluated for CD8+ T cell responses by IFN-γ ELISpot cross-sectionally after the first (**Figure 1D**) and second (**Figure 1E**) vaccine doses (25 and 24 persons respectively). This analysis revealed results consistent with the detailed longitudinal evaluations. By comparison, cross-sectional evaluation of recently COVID-19-recovered persons exhibited more stable anti-S CD8+ T cell responses over a similar time span (**Figure 1F**). These results overall confirmed that the frequency of detectable anti-spike CD8+ T cells elicited by mRNA vaccination is very short-lived, and that these kinetics differ from natural infection and likely other vaccine types.

### Evaluations by intracellular cytokine staining of both CD4+ and CD8+ T cell responses by elicited by mRNA vaccination against SARS-CoV-2 similarly reveal short-lived detection of CD4+ T cell responses

**Figure 2:**
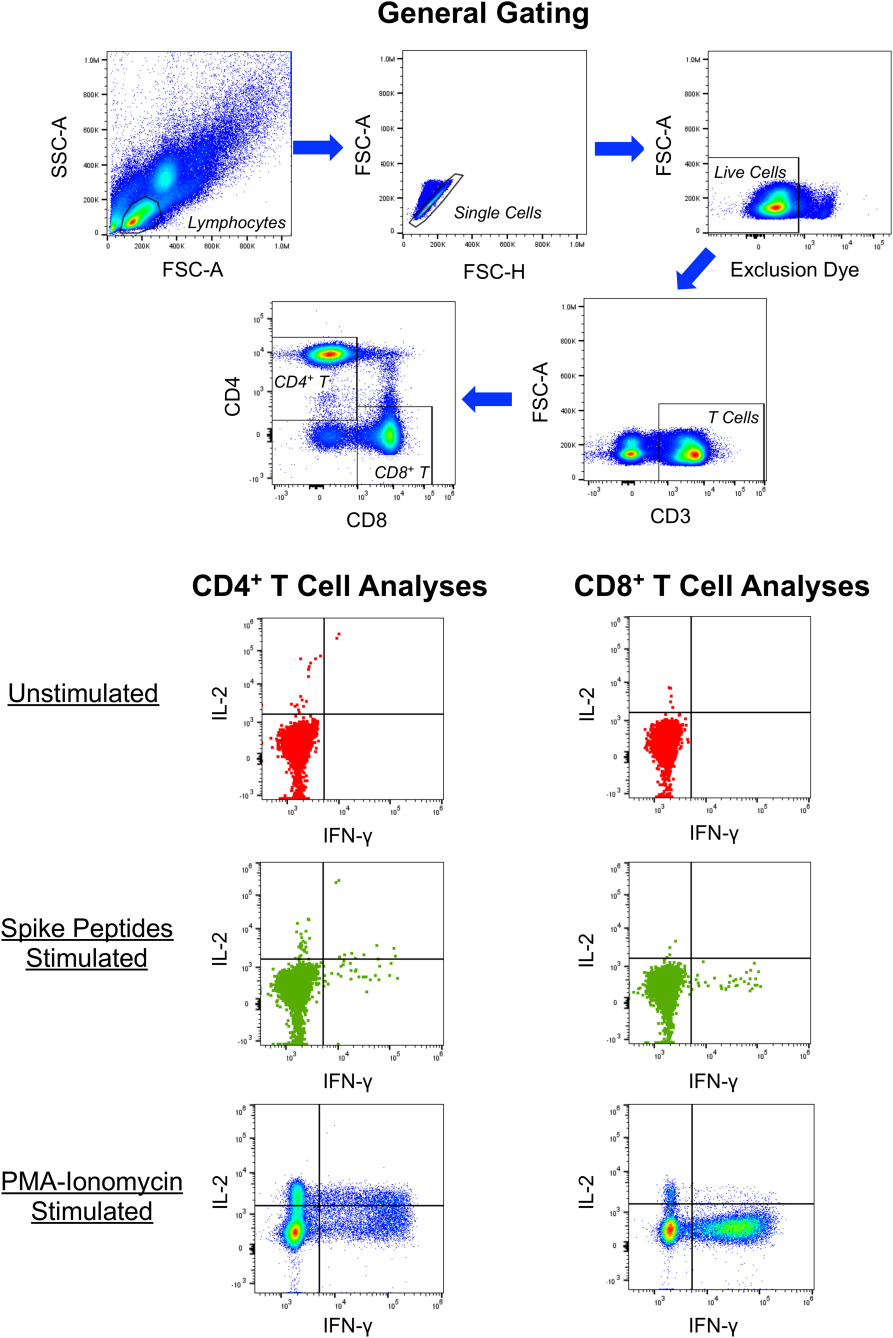
Example of intracellular cytokine staining for CD4^+^ and CD8^+^ T cell responses against SARS-CoV-2 spike. PBMC from a person 13 days after symptom onset of COVID-19 were cultured in the absence or presence of overlapping 15-mer synthetic peptides spanning the SARS-CoV-2 spike protein and assessed for production of IFN-γ, IL-2, IL-10 (not shown) and IL-4 (not shown) by intracellular cytokine staining and flow cytometry. Controls included cells cultured without peptides and PMA-ionomycin stimulated cells.

**Figure 3:**
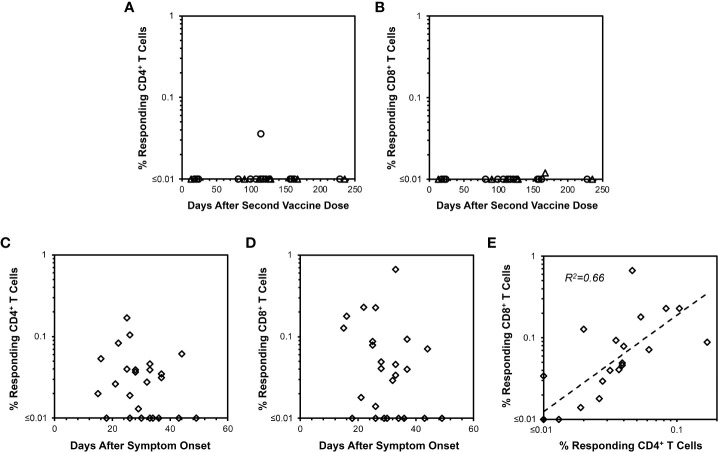
CD4+ and CD8+ T cell responses against spike measured by IFN-γ intracellular cytokine staining after mRNA SARS-CoV-2 vaccination versus natural infection. Background-subtracted values are plotted for CD4+ and CD8+ T cell spike-specific IFN-γ productiondetermined as shown in **Figure 2**.  **(A)** CD4+ T cell responses are plotted for 22 persons vaccinated with BNT162b2 (18 points from 16 persons, circles) or mRNA-1273 (7 points from 6 persons, triangles). Time points ranged from 13 to 235 days after the second vaccine dose. Only one response was detectable above 0.01% frequency. **(B)** CD8+ T cell responses measured in parallel are plotted for the same 22 persons in **(A)** Only one response was detectable above 0.01% frequency. **(C)** CD4+ T cell responses are plotted for 25 COVID-19-recovered persons ranging from 15 to 49 days after symptom onset. 17/25 (68.0%) had responses greater than 0.01%. **(D)** CD8+ T cell responses are plotted for the same 25 persons in **(C)** Again, 17/25 (68.0%) had responses greater than 0.01%. **(E)** The frequencies of responding CD4+ and CD8+ T cells from **(C, D)** are compared, demonstrating Pearson correlation r2 = 0.66, p<0.00001.

To further confirm the ELISpot findings and extend analyses to CD4+ T cells, peptide-stimulated intracellular IFN-γ staining was performed (**Figure 2**) to assess anti-spike responses on vaccinees cross-sectionally. By this assay, minimal CD4+ and CD8+ T cell responses were detectable from 13 and 235 days after completing vaccination (**Figures 3A, B**), consistent with the above ELISpot assay results on CD8+ T cells alone. These findings extended the finding of short-lived detection of T cell responses to spike after mRNA vaccination to the CD4+ T cell compartment as well, with both CD8+ and CD4+ T cell responses falling below a detectable frequency of 0.01% within days after vaccinations.

### Intracellular cytokine staining also reveals longer-lived CD4+ and CD8+ T cell responses from natural infection compared to mRNA vaccination

Evaluation of COVID-19-recovered persons by intracellular cytokine staining was performed for comparison to mRNA vaccination. In contrast to mRNA vaccination, both CD4+ and CD8+ T cell responses against spike were readily observable up to 50 days after symptom onset in COVID-19-recovered persons with relative stability over this time span (**Figures 3C, D**). The magnitudes of anti-spike CD4+ and CD8+ T cell responses correlated positively (**Figure 3E**). Simultaneously assayed anti-spike T cells producing IL-4 or IL-10 were minimal for vaccinees (**Supplementary Figures 1, 2**), whereas several COVID-19-recovered persons exhibited IL-4 but not IL-10 responses (**Supplemental Figure S3**) of unclear significance. Overall, these findings confirmed that cellular immune responses elicited by COVID-19 were more persistent compared to those from mRNA vaccination.

### Capacity to detect vaccine-elicited anti-spike memory T cell responses by culture of PBMC with lipid nanoparticle mRNA spike vaccine *in vitro*

**Figure 4:**
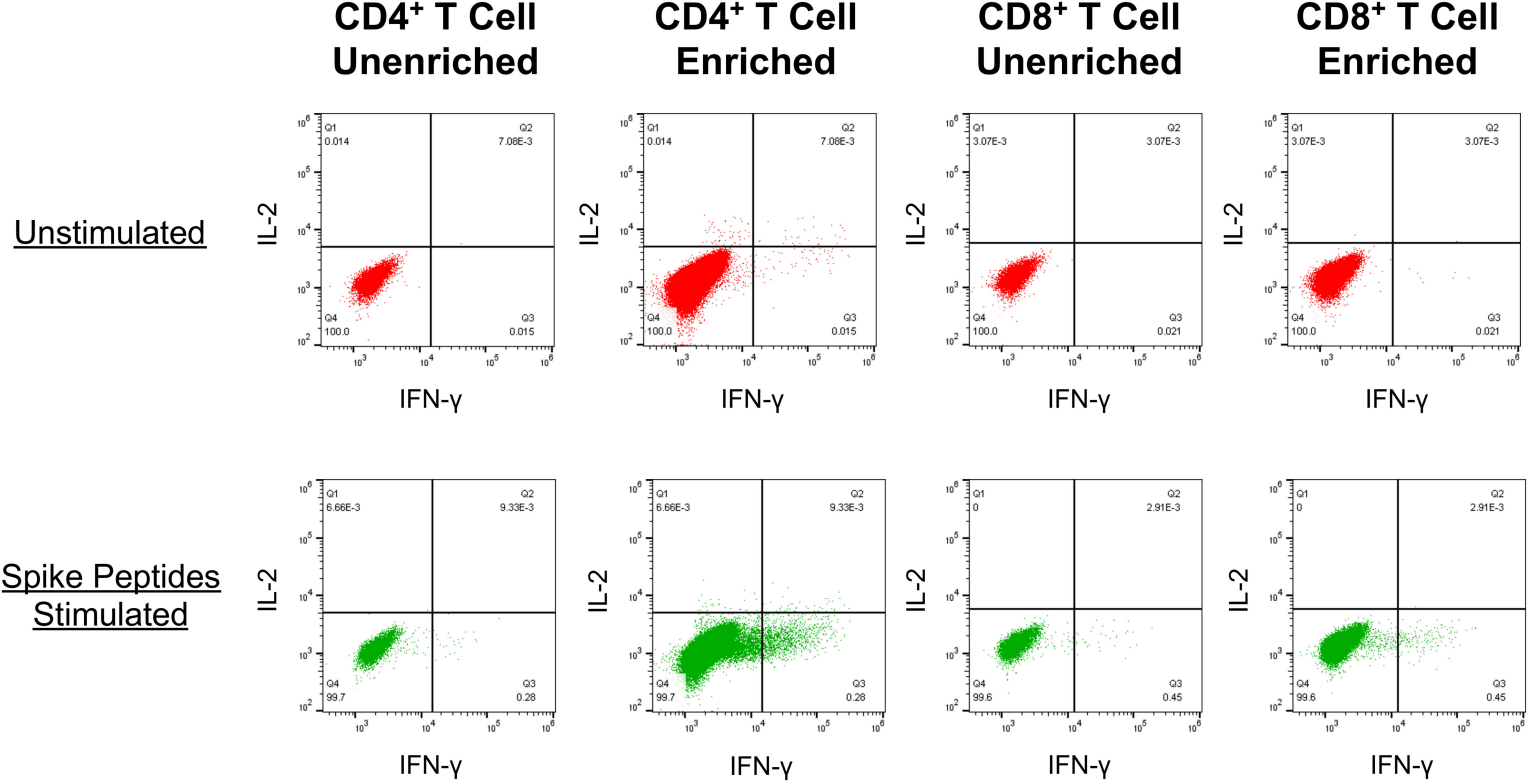
PBMC cultured with the mRNA-1273 vaccine in vitro reveal enrichment of spike-specific memory CD4+ and CD8+ T cell responses . An example is shown for detection of spike-specific T cells (as described in **Figure 2.**) in PBMCs from a SARS-CoV-2-naïve person who had completed vaccination with mRNA-1273 128 days prior. Top row: The PBMC were directly tested for T cell reactivity against spike. Bottom row: Prior to testing, the PBMC were cultured with the addition of mRNA-1273 vaccine for 14 days before testing for spike-specific T cells.

To investigate whether the fall of vaccine-elicited spike-specific T cell responses below detection indicated the absence of immune memory, we developed a novel assay for enriching memory T cells against SARS-CoV-2 spike (**Figure 4**). Conditions were established showing that *in vitro* culture of PBMCs with the mRNA-1273 vaccine at an optimal concentration of 125 mg/ml mRNA-1273 vaccine maximized expansion of memory T cells targeting spike-specific T cells, after which they could be readily detected by intracellular cytokine staining for IFN-γ (**Supplemental Figure S4**). Lower concentrations resulted in less enrichment, while higher concentrations were toxic. The results demonstrated the capacity of this assay to enrich low frequency memory T cell responses against spike in PBMC to be readily detectable.

### Despite being undetectable in standard IFN-γ-based assays, vigorous mRNA vaccine-elicited T cell memory responses against spike persist for months after vaccination

**Figure 5:**
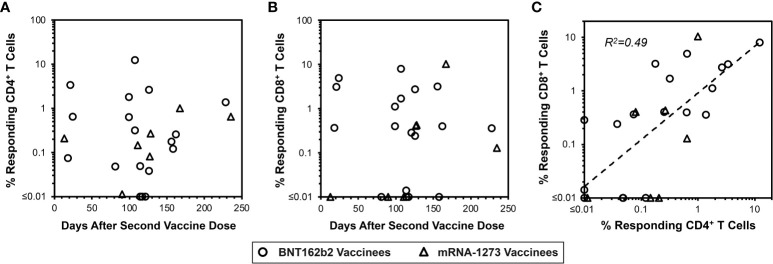
Vaccine-elicited spike-specific memory CD4+ and CD8+ T cells are persistent. In parallel to Figure 3 panels A and B, the same PBMC from 22 vaccinees were assessed for spike-specific T cell memory responses as shown in **Figure 4**. **(A)** 22/25 (88.0%) vaccinees had detectable spike-specific CD4+ T cell memory responses of greater than 0.01% frequency (14/18 BNT162b2 vaccinees, circles, and 7/7 mRNA-1273 vaccinees, triangles). **(B)** 18/25 (76.0%) vaccinees had detectable CD8+ T cell memory responses greater than 0.01% frequency (15/18 BNT162b2 vaccinees, circles, and 4/7 mRNA-1273 vaccinees, triangles). **(C)** The frequencies of spike-specific memory CD4+ and CD8+ T cells after in vitro enrichment are compared, demonstrating Pearson correlation r2 = 0.49, p<0.0001.

Given the above-noted overall lack of directly detectable responses in vaccinees 13 to 235 days after completed vaccination (**Figures 3A, B**), the memory T cell assay described above was utilized using the same PBMC samples. This evaluation demonstrated detectable spike-specific CD4^+^ and CD8^+^ T cell responses detected by IFN-γ production after culturing with mRNA-1273 for the majority of persons (**Figures 5A, B**). These memory responses generally correlated between the CD4^+^ and CD8^+^ T cell compartments (**Figure 5C**). Parallel analysis for spike-specific IL-4 and IL-10 production revealed minimal enrichment by culturing with mRNA-1273 vaccine (**Supplementary Figures 1C, D and 2C, D**). In sum, these findings confirmed vigorous persisting mRNA vaccine-elicited memory T cell responses against spike despite their lack of detection in standard IFN-γ-based T cell assays.

## Discussion

Study of the durability of antiviral immune responses after vaccination in general has mostly centered on antibodies, and has been observed to vary drastically for different vaccines and pathogens. In one study comparing several common vaccines, antibody half-lives ranged from 11 years for tetanus to more than 200 years for measles (68). The determinants of humoral immune durability are not entirely clear, but durability may relate to the vector (69–71) or vary by the target antigen itself (72, 73), and may be affected by factors such as cross-reactivity with other antigens that act to restimulate memory (74). For COVID-19 vaccines, the majority of studies have observed vaccine-elicited antibodies declining to low levels over weeks to months. Because infection-elicited anti-spike antibodies also decline rapidly after recovery from SARS-CoV-2 infection, it is likely that this reflects an intrinsic property of the spike protein rather than the mode of vaccine delivery. Given the rapid decline of protective antibodies for other common human coronaviruses and susceptibility to reinfection within months (75), this is not surprising and may be a shared property of coronaviruses.

The durability of antiviral cellular immunity, particularly CD8^+^ T lymphocytes (CTLs), is far less well defined. Accessing the human leukocyte antigen class I pathway generally has required using live vaccines such as vaccinia. Given the eradication of smallpox and cessation of vaccinia vaccination, vaccinia reactivity has been studied to address the issue of cellular immune memory. While antibody responses against vaccinia appear to be stable for many decades after vaccination (76), the cellular immune response including CTLs appears to wane to undetectable levels by sensitive ELISpot assays within about two to three decades (77–79). However, *in vitro* enrichment assays using vaccinia stimulation of PBMC demonstrated durable memory lasting five decades or more (77, 80). The degree to which memory detected in this manner would be protective against infection is unknown, although evaluations of vaccinees during smallpox outbreaks have suggested that protection may persist for many decades or life (81–83).

In comparison to vaccinia, our findings demonstrate strikingly rapid waning of mRNA vaccine-generated circulating spike-specific CTL to undetectable levels within days, not decades. In comparison, we observe that infection-generated spike-specific responses decay more gradually over months (61), which may explain why some have observed CTL responses after infection but failed to find them in COVID-naïve vaccinees (38). The observation that anti-spike memory can be detected after using mRNA-1273 vaccine to enhance responses in PBMC parallels analogous findings that vaccinia can be used to enhance memory responses that are otherwise below the limit of detection by IFN-γ ELISpot (77, 80).

Our methodology for detecting memory T cell responses against SARS-CoV-2 spike protein is novel for its use of the mRNA-1273 as an *in vitro* stimulus, but the general strategy of antigen-specific stimulation to enrich memory T cells for ELISpot detection has been utilized widely. As mentioned above, vaccinia infection of PBMC has been employed to reveal memory responses against vaccinia (77, 80), and this approach has been applied for other indications typically using small peptide antigens (84–87). While the generation of *de novo* T cell responses from naïve T cells rather than expansion of low-level memory responses by such protocols is a theoretical caveat to our approach, experimentally doing so purposely has been a technically challenging goal that requires dedicated enrichment and differentiation of specialized dendritic cells (88–91).

In agreement with prior studies on T cell responses to SARS-CoV-2 infection (92–94), we found persistence of responses over many months. However, our parallel evaluations of vaccine-elicited spike-specific T cell responses showed rapid decay to undetectable levels (by IFN-γ ELISpot) shortly after vaccination but persistence as detectable memory after spike-specific *in vitro* enrichment. In contrast to this finding, Goel et al. found an early contraction phase of the T cell response over the first three months after vaccination, with CD4^+^ and CD8^+^ T cell responses having half-lives of 47 and 24 days respectively (60). Methodologic differences likely contribute to these discordant results; they measured responses using activation markers in only the memory T cell subset, while we evaluated IFN-γ production in the total T cell population. Additionally, they assumed a steady decay rate using three time points around 20, 90, and 180 days after vaccination, while our analysis focused more closely on earlier time points. Our findings also contrast with those of Bonnet et al. (14), likely due to differences in methodology. As opposed to identifying cell frequencies by ELISpot or flow cytometry, they used a whole blood IFN-γ release assay to evaluate responses three and six months after vaccination and noted a decline over that time. Finally, our results are generally compatible with those of Lozano-Rodriguez et al. (59). They detected both early (~4 days after vaccination) and late (~8 months after vaccination) T cell responses through cytokine production and proliferation after stimulating PBMC with an overlapping peptide pool spanning spike. Thus, they also measured *in vitro* enriched memory T cell responses. They additionally noted dropping memory over time; we did not see reduced memory over a similar time span, but our analysis was cross-sectional and theirs was longitudinal.

The reasons for our observation of extremely rapid decay of anti-spike cellular immune responses after mRNA vaccinations are unclear. In contrast to CTL responses to vaccinia (77–79) or yellow fever (95) that persist over years, overall T cell responses to natural infection decay over months (61, 62) and spike-specific responses are shorter-lived than those against nucleocapsid (61). Thus, spike targeting appears intrinsically to be relatively short-lived compared to T cell responses against other pathogens. The mRNA vaccine-induced responses are still even more remarkably short-lived than those in natural infection, suggesting that the mRNA vaccine format may additionally contribute to particularly rapid decay of T cell responses. Whether this is due to the brevity of mRNA persistence and antigen expression remains to be determined, but this would be consistent with an observation that the adenoviral Ad26.COV2.S vaccine appears to give more durable cellular responses than the mRNA BNT162b2 vaccine (40).

The clinical implications of the observed rapid drop in circulating cellular immunity to undetectable levels after mRNA vaccination are unclear. Because protection from severe illness, which is predominately mediated by cellular immunity, lasts many months after mRNA vaccination (31, 43, 51–53), the lack of detection by IFN-γ ELISpot does not indicate inadequate frequency of cellular immune memory cells. This suggests that the required frequency for protection falls below the lower limit of reliable detection by ELISpot, which is generally about 50 SFC/million cells, or 0.005%. Culture of PBMC with the mRNA-1273 vaccine demonstrates the persistence of memory for many months after vaccination. This memory enrichment assay is at best semi-quantitative and our analysis is cross-sectional, so our data do not reveal a decay rate for memory below the limit of ELISpot detection that could be utilized to estimate a protective level of memory T cells. Finally, this raises questions about the utility of commonly utilized assays of T cell responses, such as ELISpot and intracellular cytokine staining, as correlates of immunity.

In summary, we find that cellular immune responses targeting spike typically decline to low frequencies below the limit of detection of standard assays remarkably quickly after mRNA vaccination (within days), while responses elicited by SARS-CoV-2 are more persistent (months). However, culture of PBMC from vaccinees with mRNA-1273 vaccine results in consistent enrichment of detectable T cell responses at least 8 months after vaccination, indicating persistence of memory. This is consistent with clinically observed protection from severe illness that lasts several months after vaccination, and raises questions regarding the utility of common assays of T cell responses as correlates of immunity. These findings are similar to studies of vaccinia cellular immunity and protection from smallpox, although T cell responses against vaccinia decay to undetectable levels over about two decades while remaining detectable after PBMC culture with vaccinia to enrich memory responses. Overall, our results suggest that the levels of memory T cells required for protective immunity against severe COVID-19 persist at least several months despite being too low to detect by standard assays. The threshold required for protection from severe disease remains to be determined.

## Data availability statement

The raw data supporting the conclusions of this article will be made available by the authors, without undue reservation.

## Ethics statement

The studies involving human participants were reviewed and approved by Institutional Review Board of University of California Los Angeles. The patients/participants provided their written informed consent to participate in this study.

## Author contributions

Overall study conceptualization: OY. Study design: ET, CH, FI, JF, PK, KF, NT, AR, GA, OY. Conducting experiments: ET, CH, FI, LG, MH. Data analysis: ET, CH, FI, PK, SK, OY. Providing reagents: JF, SK, KF, NT, AR, GA. Primary writing of the manuscript: ET, OY. Reviewing and revising the manuscript: ET, CH, FI, MH, JF, PK, SK, KF, NT, AR, GA, OY. All authors contributed to the article and approved the submitted version.
