# Supplementary material for: Persistent memory despite rapid contraction of circulating T Cell responses to SARS-CoV-2 mRNA vaccination
Source: Front Immunol. 2023 Feb 13;14:1100594. doi: 10.3389/fimmu.2023.1100594 (PMC9968837; doi:10.3389/fimmu.2023.1100594)
Supplement: Supplementary file 1 [file DataSheet_1.pdf]

SUPPLEMENTAL FIGURES

Persistent Memory Despite Rapid Contraction of Circulating T Cell Responses to SARS-CoV-2

Ellie Taus[1], Christian Hofmann[2], F. Javier Ibarrondo[2], Laura S. Gong[3], Mary Anne Hausner[2], Jennifer A. Fulcher[2], Paul Krogstad[1,4], Scott G. Kitchen[2], Kathie G. Ferbas[2], Nicole H. Tobin[4], Anne W. Rimoin[5], Grace M. Aldrovandi[4], Otto O. Yang[2,3]*

[1]Department of Molecular and Medical Pharmacology, David Geffen School of Medicine, University of California, Los Angeles, Los Angeles, CA, USA

[2]Department of Medicine, David Geffen School of Medicine, University of California Los Angeles, Los Angeles, CA, USA

[3]Department of Microbiology, Immunology, and Molecular Genetics, David Geffen School of Medicine, University of California Los Angeles, Los Angeles, CA, USA

[4]Department of Pediatrics, David Geffen School of Medicine, University of California Los Angeles, Los Angeles, CA, USA

[5]Fielding School of Public Health, University of California Los Angeles, Los Angeles, CA, USA

*** Correspondence:**
Otto Yang, BSRB 173, 615 Charles E Young Drive South, Los Angeles, CA 90095
oyang@mednet.ucla.edu

Keywords: COVID-19 Vaccine, mRNA Vaccine, SARS-CoV-2, Cellular Immunity, T cells, ELISpot, Intracellular Cytokine Staining

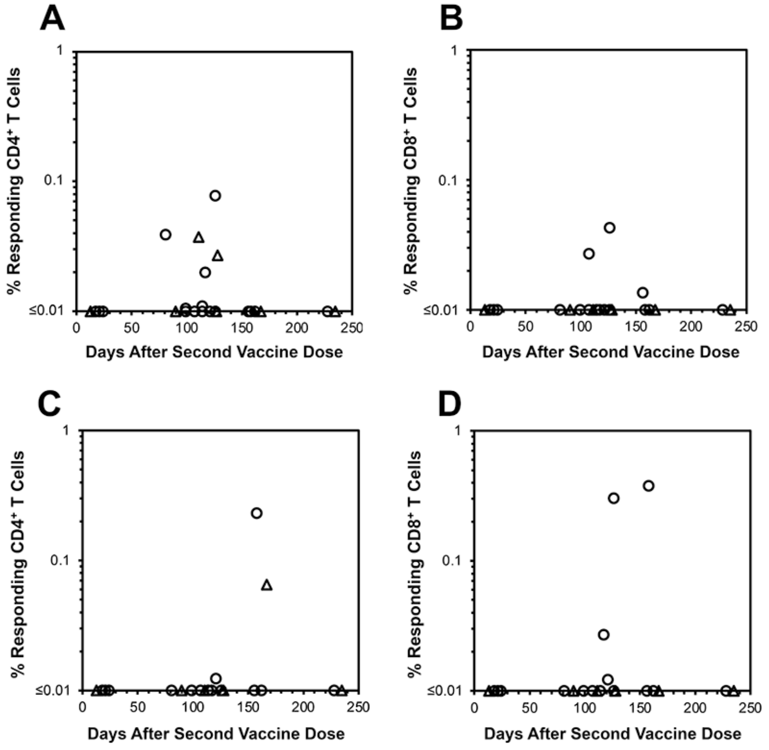

Supplemental Figure S1. Vaccine-elicited CD4^{+} and CD8^{+} T cell responses against SARS-CoV-2 spike measured by IL-4 intracellular cytokine staining. PBMC from SARS-CoV-2-naïve persons after mRNA vaccination were assessed by intracellular cytokine staining flow cytometry for CD4^{+} and CD8^{+} T cell IL-4 production in response to overlapping peptides spanning spike in parallel with Figure 3, and background-subtracted values are plotted. Data are plotted for 22 persons vaccinated with BNT162b2 (18 points from 16 persons, circles) or mRNA-1273 (7 points from 6 persons, triangles). Time points ranged from 13 to 235 days after the second vaccine dose. Panels A and B: PBMC responses in the CD4^{+} (A) and CD8^{+} (B) T cell compartments are plotted. Panels C and D: PBMC responses after *in vitro* enrichment in culture with mRNA-1273 as performed in parallel with Figure 5 are shown for the CD4^{+} (C) and CD8^{+} (D) T cell compartments.

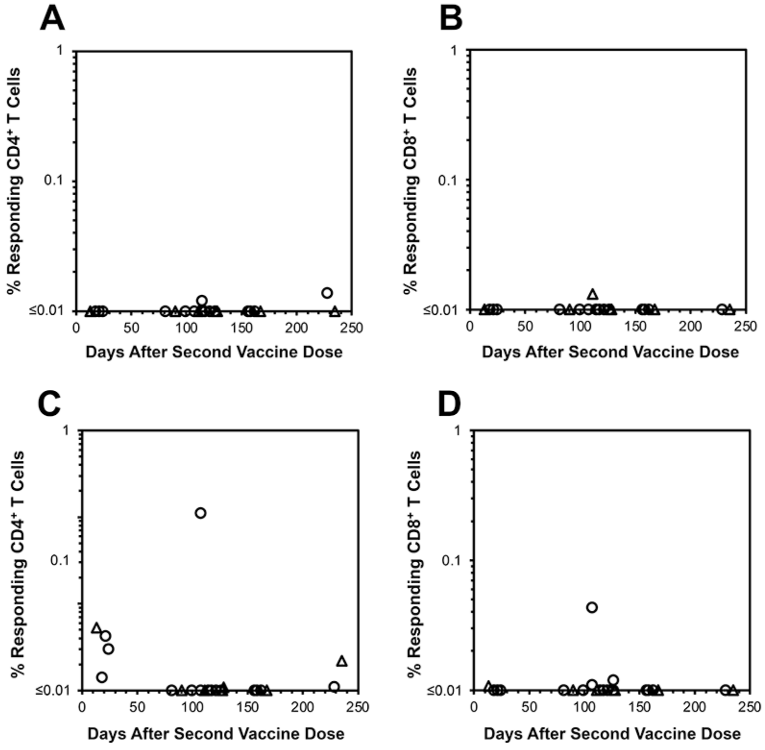

Supplemental Figure S2. Vaccine-elicited $CD4^+$ and $CD8^+$ T cell responses against SARS-CoV-2 spike measured by IL-10 intracellular cytokine staining. PBMC from SARS-CoV-2-naïve persons after mRNA vaccination were assessed by intracellular cytokine staining flow cytometry for $CD4^+$ and $CD8^+$ T cell IL-10 production in response to overlapping peptides spanning spike in parallel with Figure 3, and background-subtracted values are plotted. Data are plotted for 22 persons vaccinated with BNT162b2 (18 points from 16 persons, circles) or mRNA-1273 (7 points from 6 persons, triangles). Time points ranged from 13 to 235 days after the second vaccine dose. Panels A and B: PBMC responses in the $CD4^+$ (A) and $CD8^+$ (B) T cell compartments are plotted. Panels C and D: PBMC responses after *in vitro* enrichment in culture with mRNA-1273 as performed in parallel with Figure 5 are shown for the $CD4^+$ (C) and $CD8^+$ (D) T cell compartments.

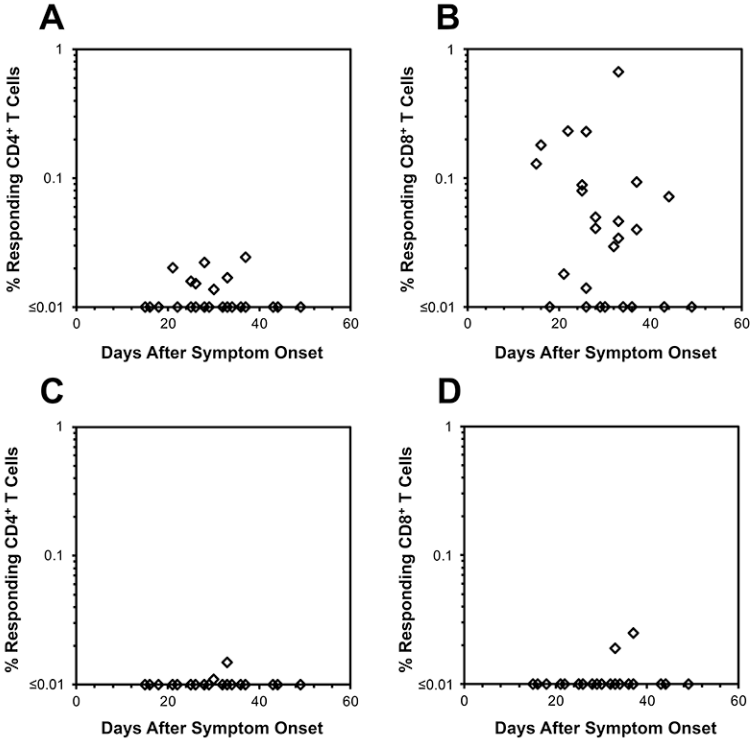

Supplemental Figure S3. CD4$^+$ and CD8$^+$ T cell responses against spike measured by IL-4 and IL-10 intracellular cytokine staining in persons after COVID-19. PBMCs from 25 COVID-19-recovered persons were assessed by intracellular cytokine staining flow cytometry for CD4$^+$ and CD8$^+$ T cell IL-4 and IL-10 production in response to overlapping peptides spanning spike in parallel with Figure 3, and background-subtracted values are plotted. Panels A and B: IL-4 responses are plotted for CD4$^+$ (A) and CD8$^+$ T (B) cells. Panels C and D: IL-10 responses are plotted for CD4$^+$ (C) and CD8$^+$ T (D) cells.

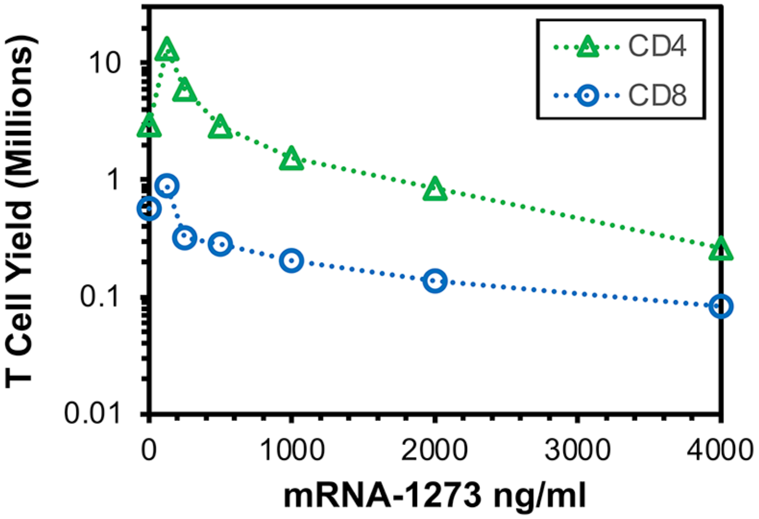

Supplemental Figure S4. Titration of mRNA-1273 to enrich for memory T cell responses against spike in vaccinees. PBMCs from a SARS-CoV-2-naïve who had completed the two dose vaccination series with the BNT162b2 vaccine six months prior were cultured for 14 days with varying concentrations of mRNA-1273 vaccine, starting with 2 x 10^6 cells per condition. Total cell counts and flow cytometry for fractions of $CD4^+$ and $CD8^+$ T cells were then obtained to calculate the yields of these cells. The highest yield was seen for 125 ng/ml added vaccine, with 13.3 x 10^6 $CD4^+$ T cells and 0.9 x 10^6 $CD8^+$ T cells, compared to control cells without stimulus that yielded 3.0 x 10^6 $CD4^+$ T cells and 0.6 x 10^6 $CD8^+$ T cells. A parallel positive control stimulation with an anti-CD3 monoclonal antibody yielded 2.3 x 10^6 $CD4^+$ T cells and 20.0 x 10^6 $CD8^+$ T cells (not plotted), a different pattern of relative expansion of $CD4^+$ versus $CD8^+$ T cells, supporting the specificity of stimulation with mRNA-1273. Evaluation of the resulting cells by IFN-γ ELISpot assays for spike were performed using 2 x 10^5 cells per well (as described in the Methods) on cells stimulated 125 ng/ml, 250 ng/ml, and 500 ng/ml of mRNA-1273. All of these mRNA-1273-stimulated wells had too many spots to quantify at >400 SFC/well (>2000 SFC/million cells) although the well with cells stimulated by 125 ng/ml appeared the most saturated; data not shown.
